# Maximum Likelihood Inference of Small Trees in the Presence of Long Branches

**DOI:** 10.1093/sysbio/syu044

**Published:** 2014-07-04

**Authors:** Sarah L. Parks, Nick Goldman

**Affiliations:** European Molecular Biology Laboratory, European Bioinformatics Institute (EMBL-EBI), Wellcome Trust Genome Campus, Hinxton, CB10 1SD, United Kingdom

## Abstract

The statistical basis of maximum likelihood (ML), its robustness, and the fact that it appears to suffer less from biases lead to it being one of the most popular methods for tree reconstruction. Despite its popularity, very few analytical solutions for ML exist, so biases suffered by ML are not well understood. One possible bias is long branch attraction (LBA), a regularly cited term generally used to describe a propensity for long branches to be joined together in estimated trees. Although initially mentioned in connection with inconsistency of parsimony, LBA has been claimed to affect all major phylogenetic reconstruction methods, including ML. Despite the widespread use of this term in the literature, exactly what LBA is and what may be causing it is poorly understood, even for simple evolutionary models and small model trees. Studies looking at LBA have focused on the effect of two long branches on tree reconstruction. However, to understand the effect of two long branches it is also important to understand the effect of just one long branch. If ML struggles to reconstruct one long branch, then this may have an impact on LBA. In this study, we look at the effect of one long branch on three-taxon tree reconstruction. We show that, counterintuitively, long branches are preferentially placed at the tips of the tree. This can be understood through the use of analytical solutions to the ML equation and distance matrix methods. We go on to look at the placement of two long branches on four-taxon trees, showing that there is no attraction between long branches, but that for extreme branch lengths long branches are joined together disproportionally often. These results illustrate that even small model trees are still interesting to help understand how ML phylogenetic reconstruction works, and that LBA is a complicated phenomenon that deserves further study. [analytic solutions; long branch attraction; maximum likelihood; simulation.]

Amongst the methods for phylogenetic tree reconstruction from molecular sequence data, maximum likelihood (ML) is one of the most popular due to its statistical basis, robustness, and the fact that it appears to suffer less from biases. Additionally, ML is known to be a consistent method if the assumed model is correct (Chang 1996; Rogers 1997), meaning that as the amount of data tends to infinity the probability of obtaining the correct tree tends to one. Consistency, however, is not informative about performance of a method with finite data, and with finite data ML can struggle, particularly if long branches are present on the tree. The reasons for this are unknown. ML with the correct model should be able to deal with parallel substitutions and multiple substitutions at sites (Chang 1996), phenomena that occur when branches are long, but despite this it has been reported to be biased toward trees with long branches placed together (Huelsenbeck 1995).

One of the reasons that biases in ML reconstruction (e.g., issues caused by long branches) are not well understood is that very few analytical solutions for ML exist, and the solutions that do exist are for small trees and simple models. This means that ML tree reconstruction is generally carried out using numerical maximization and heuristics. Yang (2000) derived a set of analytic solutions for a three-taxon tree using two-state characters. Since then further analytic solutions for three-taxon trees with two-state or four-state characters, and four-taxon trees with two-state characters have been derived (Chor et al. 2001, 2006a, 2006b; Chor and Snir 2004, 2007). All of these studies consider trees with a molecular clock, meaning that biases caused by long-tip branches cannot be studied, as it is not possible to have short tip branches joined to long-tip branches. Further, analytical solutions are required to fully understand long branch biases.

Long branches represent a large amount of evolutionary change for which there are only a few observations. Various effects of long branches on tree reconstruction have been reported, starting with Felsenstein (1978). Felsenstein studied a four-taxon tree with two long branches (P) and three short branches (Q) (Fig. 1). He proved that with two-state characters there are combinations of P and Q for which parsimony reconstruction is inconsistent. This region of branch length space is now widely called the Felsenstein zone (Huelsenbeck and Hillis 1993). Since Felsenstein's paper, conditions for inconsistency of parsimony have been extended to any number of character states and five different parameters for branch lengths instead of two (Zharkikh and Li 1992; Schulmeister 2004). Larger trees have also been examined, with further inconsistency conditions found (Kim 1996).

**Figure 1.**
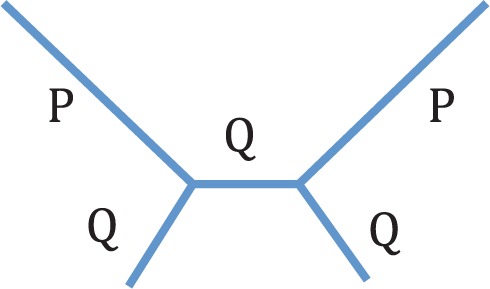
Tree used by Felsenstein to show that parsimony could be inconsistent. The short branch length is Q and the long branch length is P.

Following Felsenstein's early work on inconsistency it became widely accepted that such problems were due to “attraction” amongst long branches. It also became clear that these problems may not be restricted to parsimony only. Numerous simulation studies tested whether the accuracy of other tree reconstruction methods is affected by the presence of two long branches (Huelsenbeck and Hillis 1993; Kuhner and Felsenstein 1994; Gaut and Lewis 1995; Huelsenbeck 1995). One of the most thorough studies was carried out by Huelsenbeck (1995). Using the same tree as Felsenstein, but with four-state characters, he tested the consistency, efficiency, and robustness of 26 reconstruction methods. This showed that under model misspecification all methods could suffer from inconsistency, and that long branch effects seem to be more of a problem with shorter sequences. It also showed that the presence of long branches does seem to affect ML, although the effects were not as strong as for the other methods investigated.

The term “Long Branch Attraction” (LBA) has become widely used to describe long branches being incorrectly placed together on a phylogenetic tree. However, LBA is not well defined and statistical inconsistency, model violation, and claims that certain methods are unable to deal with parallelism and convergence have been variously cited as both definitions and explanations (Philippe and Laurent 1998; Sanderson et al. 2000; Anderson and Swofford 2004). Initial studies on LBA were theoretical, with data obtained by simulation. However, after the coining of the term LBA by Hendy and Penny (1989), there was interest in whether it could affect real data. Conclusive biological evidence has been difficult to find because the true tree is never known for real data. However, the publication of a number of papers proposing that LBA can affect real data (Huelsenbeck 1997, 1998) led to LBA being frequently cited as the reason for unexpected phylogenetic results (Stiller and Hall 1999; Sanderson et al. 2000; Philippe and Germot 2000; Wiens and Hollingsworth 2000; Qiu et al. 2001; Omilian and Taylor 2001; Dacks et al. 2002; Stefanović et al. 2004; Wilcox et al. 2004; Inagaki et al. 2004; Fares et al. 2006; Barros et al. 2008; Dabert et al. 2010; Bodilis et al. 2011). Methods to detect LBA have also been widely discussed and include: finding two long branches together; showing a better method does not place the long branches together; showing the branches are long enough to attract by simulation; breaking up a long branch; and removing one of the long branches and reconstructing the tree to see if the other long branch moves (Huelsenbeck 1997; Bergsten 2005). There is, however, no method that can guarantee a particular topology has been caused by LBA.

In addition to being poorly defined and difficult to locate, the reasons for assuming problems to arise from interactions between multiple long branches, or for naming LBA an “attraction”, are not clear. “Attraction” implies that there is an interaction between long branches and that this interaction causes them to be placed closer together. However, this has never been proven and indeed our knowledge of the problems engendered by long branches is incomplete. In this article, we aim for a greater understanding of the behavior of ML tree inference in the presence of individual long branches. We then extend our analysis to the case of two long branches, looking for any additional effects related to their interaction. To do this we need to distinguish between difficulty in placing long branches and attraction between long branches. If an attraction were to exist then its effects could be interpreted, and hence measured, in different ways. We will define two such ways as “long branch joining” (LBJ) where long branches are incorrectly joined together on a tree, and “long branch closeness” (LBC) where long branches are closer together on the reconstructed topology than on the true topology. Knowledge of whether either of these two phenomena occur will lead to a greater understanding of the effects of long branches on tree reconstruction. We will focus on ML with the correct model, which is consistent. We find this more approachable than looking at model misspecification: with the wrong model anything could happen, but under the correct model ML is expected to perform well.

In this article, we start by looking at the placement of one long branch by ML. This is important because correct placement of a branch between two nodes is necessary for all tree reconstruction. We use a three-taxon tree as it is the simplest possible tree for reconstruction yet gives interesting and counterintuitive results. Placement of long branches is assessed by simulations followed by ML tree reconstruction for the simulated data sets. The distribution of placement of long branches is then studied using analyses of both ML and distance matrix (DM) equations for three-taxon trees. This gives insight into why long branches may cause problems for tree reconstruction, and allows for partial analytical solutions of the four-state character, three-taxon tree without a molecular clock. We then use knowledge about the placement of one long branch to look at the effect of two long branches. Four-taxon trees are used, as the three possible topologies are the simplest that allow us to investigate both LBC and LBJ phenomena. We test for the existence of both LBC and LBJ, allowing us to split any potential “attraction” into two parts and see which occur. This reveals the complexity of the problem and highlights that further work will be necessary to fully understand it.

## Methods

### Evolutionary Models and Trees

This article considers nucleotide sequences evolved under Jukes Cantor (JC) evolution (Jukes and Cantor 1969; Yang 2006). This is both the simplest model and shows the properties of ML estimation on which we wish to concentrate. Sequences are simulated without insertions or deletions so no alignment of the sequences is necessary. It is assumed that each site in the alignment evolves independently and at the same rate. Data at different sites are thus assumed to be independent and identically distributed. Therefore, the order of the sites does not matter, just the counts of each possible nucleotide pattern. Unrooted trees are used throughout this article as JC is reversible and no molecular clock is assumed; hence a rooted tree cannot be found.

**Figure 2.**
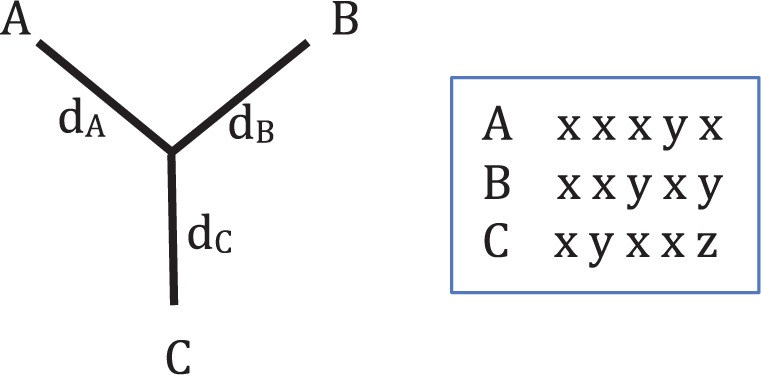
Unrooted three-taxon tree with the five possible site patterns when considering JC evolution, where *x*, *y*, and *z* are any three different nucleotides.

For an unrooted three-taxon tree (Fig. 2) there are 4^3^ = 64 possible combinations of the nucleotides at a site over the three taxa. These combinations are called site patterns. In the JC model, each nucleotide has equal base frequency and mutation rate, meaning that many of these site patterns have the same probability of occurring. In fact it does not matter which nucleotides are present for different taxa, just whether the nucleotides are different for the different taxa. This means that the site patterns can be reduced to just five patterns of interest, *P* = {*xxx*,*xxy*,*xyx*,*yxx*,*xyz*}, where *x*, *y*, and *z* are any three different nucleotides. The pattern *xxx* thus represents four possible nucleotide combinations (AAA, CCC, GGG, and TTT), and the remaining patterns represent 12, 12, 12, and 24 nucleotide combinations, respectively. Data can then be represented as counts of these five different patterns from a sequence alignment. For an alignment of length *n*, these counts will be written as *n*_*r*_ for each pattern *r* ∊ *P*, and 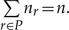 For a four-taxon tree there are 256 possible site patterns, which can be reduced to 15 patterns of interest for JC evolution.

### Maximum Likelihood

To look for analytical solutions, the likelihood function was derived for a three-taxon tree using standard methods (Yang 2006). This derivation is shown in the Supplementary Methods available on Dryad (http://dx.doi.org/10.5061/dryad.rp7qv).

ML tree reconstruction was also conducted using the baseml program from the PAML package (Yang 2007). As we investigate small trees we can perform a heuristic search for the ML branch lengths for each topology individually and then compare to find the ML tree. Use of a heuristic search means that results may be dependent on the starting values used for branch lengths. Additionally the presence of long branches makes the search more difficult. To improve our ability to find ML values, baseml was run from five different starting points for each analysis, and the ML tree was chosen as the tree with the highest likelihood from these runs. To check that five runs was enough we have assessed how often the results would change if only four runs were carried out. The changes were minimal, even for long branch lengths. Baseml was modified to help it find the ML tree when the likelihood was very flat, and to make sure restrictions on branch lengths did not stop it from finding the ML tree. Details on how to make these modifications can be found in the PAML documentation. If runs of baseml found trees with different long branch lengths but a very similar likelihood, we hypothesized that the ML tree in fact had an infinite branch length. This was then tested by analytically calculating the likelihood of the tree with an infinite branch length and comparing it with the likelihoods from baseml. A higher analytical likelihood was taken as confirmation that the branch was infinitely long. In this case there is no information about where the branch should be placed on the tree, so any placement made by baseml would be artifactual. Therefore, for these trees the branch in question was recorded as being of infinite length and having no meaningful position on the tree.

To test our procedures for artifacts, phylogenetic inferences were repeated using PhyML (Guindon et al. 2010). Our modified version of baseml invariably found either the same tree as PhyML or a tree with a higher likelihood, increasing our confidence in baseml's ML estimates for the analyses needed in this article. Since baseml and PhyML are optimized for different tasks in phylogenetic inference, we do not draw any broader conclusions about the merits of the two programs.

### DM Equations

DM methods for inferring phylogenetic trees are based on computing pairwise distances and using some criterion to fit these distances to a tree (Yang 2006). Although we do not study performance of DM methods in this article, we find it useful to draw on some of these ideas to help understand the performance of ML methods. Under the JC model, the pairwise distance is 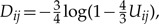 where *U*_*ij*_ is the fraction of bases that differ between the two taxa *i* and *j* (Yang 2006). For each pair of taxa, *U*_*ij*_ can be written as a sum of pattern counts divided by the sequence length; for example, between taxa *A* and *B* of Figure 2, *U*_*AB*_ = (*n*_*xyz*_ + *n*_*xyx*_ + *n*_*yxx*_)/*n*. If *U*_*ij*_ ≥ 0.75, then the distance between the two taxa is infinite, so for a finite data set there is a maximum distance between two taxa that can be measured before the two taxa are estimated to be infinitely far apart.

There are a variety of methods that can be used to fit pairwise distance measures to a tree (Yang 2006). On an unrooted three-taxon tree minimum evolution, neighbor-joining and both weighted and unweighted least squares methods result in the same branch lengths, as the distances can be exactly fit to the tree. For trees with more taxa it is often not possible to fit the distances exactly, so the different methods may give different results. Here, the branch lengths are
(1)
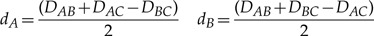

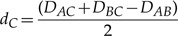

These calculations can result in negative branch lengths which are not biologically meaningful. Some software, therefore, require a positivity constraint to guarantee results that are meaningful in a phylogenetic context.

### Simulations

For three-taxon trees simulations were run under JC evolution producing 5000 data sets of 300 bp sequences, unless otherwise stated. This is a realistic sequence length for a small protein, and allows us to look at how ML works for limited data. For four-taxon trees sequence length was increased to 1000 bp due to the use of two long branches. All simulations were conducted using evolver from the PAML package (Yang 2007).

## Results and Discussion

### One Long Branch on Three-Species Trees

#### ML inference

**Figure 3.**
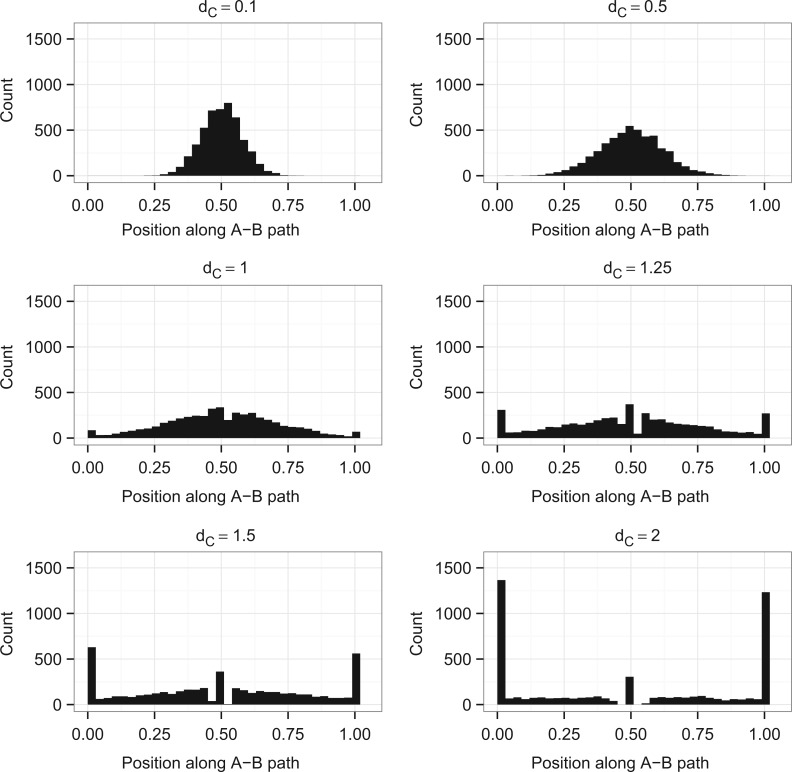
Distributions of the location of the branch leading to C on the A–B path for trees simulated with *d*_*C*_ = 0.1,0.5,1,1.25,1.5,2. For each value of *d*_*C*_, 5000 data sets were run; those that produced a tree with a predicted infinite branch length are not plotted: this corresponds to 0, 0, 0, 0, 1, and 92 data sets, respectively. The distributions of *d*_*C*_ and *d*_*A*_ + *d*_*B*_ along with plots of the position of C against *d*_*C*_ and *d*_*A*_ + *d*_*B*_ are shown in Supplementary Figures 1–4 available on Dryad; http://dx.doi.org/10.5061/dryad.rp7qv.

To explore the placement of one long branch on a tree we simulated data from a three-taxon unrooted tree (Fig. 2) with a long branch, and constructed and examined trees inferred from this simulated data. The three-taxon case is used as it is the simplest possible; there is only one topology so the only inference question is the branch lengths. Six different branch lengths were used for *d*_*C*_ (*d*_*C*_ = 0.1,0.5,1,1.25,1.5,2). So that we could concentrate on the placement of the long branch, *d*_*A*_ and *d*_*B*_ were set to 0.1 to make the distance from A to B easy to estimate (Supplementary Fig. 1 available on Dryad; http://dx.doi.org/10.5061/dryad.rp7qv). Estimation of *d*_*C*_ also behaves as expected, getting harder as *d*_*C*_ increases (Supplementary Fig. 2 available on Dryad; http://dx.doi.org/10.5061/dryad.rp7qv). Unexpected results come from looking at the position of where the branch to C joins the A–B path (Fig. 3). The placement of C is measured as a fraction along the A–B path. If C is placed on one end of the A–B path, so that the branch to A has length 0 (*d*_*A*_ = 0), then C is measured as being at 0 on the A–B path; if C is placed on the other end, and *d*_*B*_ = 0, then C is measured at 1. Trees with inferred infinite branch lengths are not included in these plots.

When *d*_*C*_ is of the same length as the other branches (*d*_*C*_ = 0.1) then tree reconstruction is accurate and C is distributed around its original position. As *d*_*C*_ increases the distribution spreads over the A–B path and, counterintuitively, starts to accumulate at the edges of the A–B path and in the centre. For long *d*_*C*_, we expected the placement of C to be uniform over the A–B path, reflecting the lack of information about the relationship between C and the other taxa, and that if there was a peak it would be gradual and centered. This was not seen here.

Note that for these simulations *d*_*A*_ and *d*_*B*_ were kept constant. The same effect is seen for other values of *d*_*A*_ and *d*_*B*_, although the precise values of *d*_*C*_ needed for the effect to become apparent depends on *d*_*A*_ and *d*_*B*_ (results not shown). The effect is also present for all finite values of *n*; as *n* increases the effect is less for any given combination of *d*_*A*_, *d*_*B*_, and *d*_*C*_ but it can again be made to appear by increasing *d*_*C*_. Supplementary Figure 5 (available on Dryad; http://dx.doi.org/10.5061/dryad.rp7qv) shows the proportion of data sets giving trees with branch lengths of zero for increasing *d*_*C*_ lengths and different sequence lengths. For a longer sequence length (*n* = 1000) the proportion of data sets giving trees with branch lengths of zero for a given value of *d*_*C*_ is lower than for *n* = 300; for a shorter sequence length (*n* = 100) it is higher. ML is, however, consistent under the correct model so for any finite *d*_*A*_, *d*_*B*_, and *d*_*C*_, as *n* → ∞ the estimates will tend toward the correct values and the effect will disappear.

Faced with the counterintuitive results of Figure 3, our next goal is to explain these distributions. First, we concentrate on the feature that when *d*_*C*_ is large many of the reconstructed trees have *d*_*A*_ = 0 or *d*_*B*_ = 0. To understand this we need to know the features of data sets that cause trees with zero branch lengths. We use DM methods as an initial approach, followed by an analysis of the ML equations. Combining these two approaches allows us to find maxima for the ML equations with zero or infinite branch lengths, and predict quite accurately when these will be global maxima. This means that for a given data set we can predict if the tree will have a zero or infinite branch length; for trees where we predict this we can also derive the branch lengths of the other branches.

#### DM analysis

The simulated data sets were analyzed using DM methods because DM equations can be easy to interpret and may give intuition into the behavior shown in Figure 3. Equation 1 gives the branch lengths of the three-taxon tree obtained using DM methods. One of the branch lengths is zero or negative if the triangle inequality is violated and one of the following conditions holds:
(2)
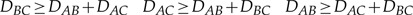


**Figure 4.**
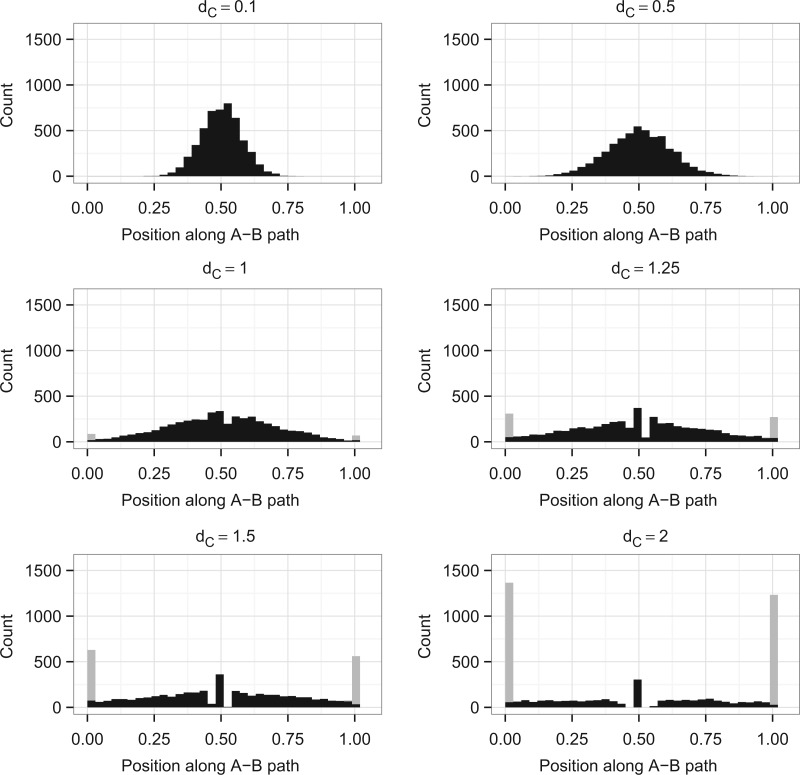
Stacked histogram showing distributions of the location of the branch leading to C on the A–B path for trees with *d*_*C*_ = 0.1,0.5,1,1.25,1.5,2. The distributions are the same as in Figure 3, but have been split so trees predicted to have zero branch lengths are colored in gray, and the remaining trees are in black. Incorrect predictions are those that are gray but not located at 0 or 1 on the *x*-axis, or black and located at 0 or 1.

To use these conditions as predictors for ML results we calculate pairwise distances for each data set from its pattern count data (as explained in “Methods” section) and check if the inequalities given above hold. If one of the inequalities holds, then one of the branch lengths is less than or equal to zero for the DM method and we predict that the branch length will be zero for ML. Figure 4 shows a version of Figure 3 where the data sets with predicted zero branch lengths are plotted in gray and the remaining data sets are in black. This shows that the accuracy of the conditions is high. Accuracy will be more fully examined later.

**Figure 5.**
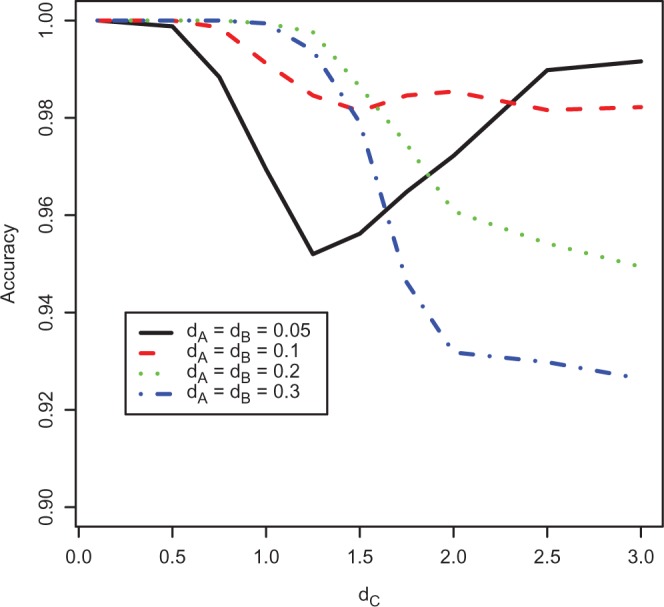
The accuracy of DM conditions for predicting zero branch lengths on ML trees for different long branch lengths. Four different lengths of A–B have been used, with *d*_*A*_ = *d*_*B*_ throughout. Accuracy is defined as the proportion of true results, i.e., the number of true positives and true negatives divided by the total number of results.

Some inferred trees have infinite branch lengths, making placement of taxon C impossible. Therefore, we are also interested in identifying trees with infinite branch lengths from DM analyses. Pairwise distances are infinite if *U*_*ij*_ ≥ 0.75 (see “Methods” section). If exactly one pairwise distance is infinite, then one of the conditions shown above (Equation 2) holds. This means that with DM methods there will be one negative branch length and two infinite branches (Equation 1). By comparing this with ML results we find that this corresponds to cases where the ML tree has one zero branch length, and finite lengths for the other branches. This can therefore be included as a case where a zero branch length is predicted if one of the conditions above (Equation 2) holds.

If two pairwise distances are infinite, for example *D*_*AC*_ and *D*_*BC*_, then there can be no knowledge about the placement of one of the taxa, here C, so the length of its branch will be infinite. So for any taxon *X*, if the other two taxa are *Y* and *Z*, then we would expect the branch to *X* to be infinite if *D*_*YX*_ and *D*_*ZX*_ are infinite. If three pairwise distances are infinite then there can be no knowledge of the relationship of any of the taxa so at least two of the branch lengths should be infinite. This gives conditions for infinite branches, which again can be used as predictors for ML results. All predictors are shown in Table 1.

**Table 1.** Predictions for branch lengths of the ML tree using pairwise distances

Conditions	Prediction
*D*_*BC*_ ≥ *D*_*AB*_ + *D*_*AC*_ (incl. *D*_*BC*_ = ∞)	*d*_*A*_ = 0
*D*_*AC*_ ≥ *D*_*AB*_ + *D*_*BC*_ (incl. *D*_*AC*_ = ∞)	*d*_*B*_ = 0
*D*_*AB*_ ≥ *D*_*AC*_ + *D*_*BC*_ (incl. *D*_*AB*_ = ∞)	*d*_*C*_ = 0
*D*_*AB*_ = ∞ & *D*_*AC*_ = ∞	*d*_*A*_ = ∞
*D*_*AB*_ = ∞ & *D*_*BC*_ = ∞	*d*_*B*_ = ∞
*D*_*AC*_ = ∞ & *D*_*BC*_ = ∞	*d*_*C*_ = ∞
*D*_*AB*_ = ∞ & *D*_*AC*_ = ∞ & *D*_*BC*_ = ∞	At least two of the branch lengths are infinite

The accuracy of these DM-based predictors of ML behavior was tested using simulation, comparing ML results with predictions made from the count data. We simulated 5000 data sets from the tree in Figure 2 with *d*_*C*_ = 0.1,0.5,1,1.25,1.5,2 and *d*_*A*_ = *d*_*B*_ = 0.05,0.1,0.2,0.3. The values for *d*_*A*_ and *d*_*B*_ were again chosen to exhibit a range of lengths where estimation would be relatively easy. In these simulations, the DM conditions for infinite branch lengths matched ML with 100% accuracy. The accuracy for the zero branch length DM conditions is shown in Figure 5. These conditions are at least 95% accurate for all simulations apart from *d*_*A*_ = *d*_*B*_ = 0.3 where they remain more than 90% accurate.

Zero-length branches can be explained by noting that with long branch lengths we frequently get data that suggest |*D*_*BC*_ − *D*_*AC*_| ≥ *D*_*AB*_. This occurs because estimates of *D*_*BC*_ and *D*_*AC*_ have high variance if *d*_*C*_ is large. This then leads to inference of a zero branch length.

The good prediction accuracy suggests that the DM conditions are closely related to ML inference. The next section attempts to derive analytic ML solutions that would give perfect understanding of our counterintuitive findings.

#### ML analysis

To derive branch lengths we need to find the global maximum of the likelihood equation. One approach to do this is to find all of the local maxima and compare their values to find the greatest. We have not been able to achieve this due to the complexity of the ML equations. However, we have been able to find all the local maxima with zero or infinite branch lengths. We can then compare the likelihoods to find the greatest, and using the DM results we can then predict when this result is the global maximum. This allows us to predict not only if there is a zero or infinite branch length, but also the other branch lengths on the tree.

**Figure 6.**
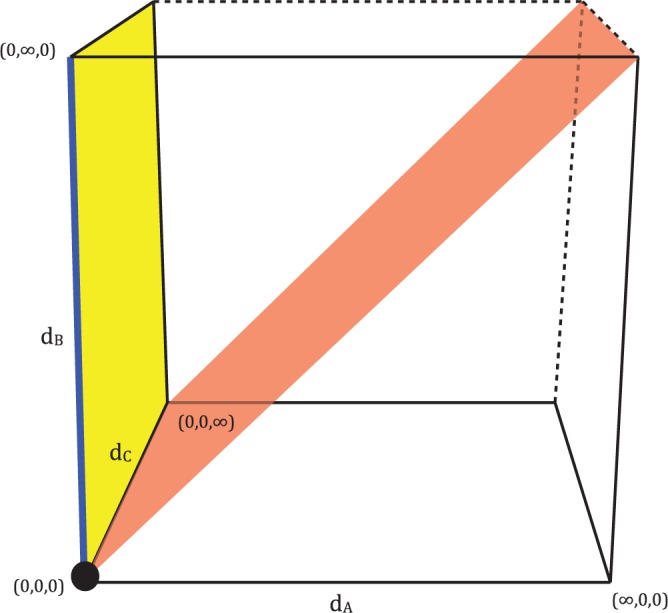
The solution space of the ML equation is an infinitely bounded convex polyhedron. One point (black), one line (blue), one surface plane (yellow), the interior plane *d*_*A*_ = *d*_*B*_ (red), and three lines where two variables are at ∞ (dotted line) are highlighted; when the ML equation is restricted to regions such as these analytical solutions can be found for local maxima.

The ML equation for a three-taxon tree is a function of the five pattern counts and the three branch lengths (see Supplementary Methods, Equation 1 available on Dryad; http://dx.doi.org/10.5061/dryad.rp7qv). Our aim is to find the three optimal branch lengths for a given set of pattern counts. The solution space of the ML equation is therefore a 3D region with each dimension representing a branch length. Branch lengths are restricted to be non negative, so the boundaries of the region occur when one or more of the branches are either zero or infinite. The space representing all solutions with any zero or infinite branch lengths is, therefore, the surface of a convex polyhedron that has been made compact (i.e., closed and bounded) by the addition of points at infinity, from now on described as a cube, giving 26 regions (8 points, 12 lines, and 6 planes) to investigate. Figure 6 illustrates this as a cube where finite boundaries have been drawn to represent ∞ for ease of understanding. The interior of the region represents all cases, where each of *d*_*A*_, *d*_*B*_, and *d*_*C*_ is positive and finite.

To solve for local maxima of the likelihood function at the boundaries, we restrict the ML equations to each of the points, lines, or planes on the surface of the cube and solve for maxima in each region. Standard methods were used to solve for maxima (Luenberger 1984); the derivations of all of the possible maxima on boundaries are shown in the Supplementary Methods (available on Dryad; http://dx.doi.org/10.5061/dryad.rp7qv). Because we have not found a solution for all maxima in the interior of the cube we cannot in general determine whether each maximum will be a local or global maximum; to do this we would have to compare the likelihood values of all the maxima, including any in the interior. However, in some special cases we are able to determine the global maximum, and these are detailed in Table 2. The rest of the local maxima are detailed in Table 3.

**Table 2.** Global maxima of the ML equations on the boundaries of the solution space

Conditions		(*d*_*A*_,*d*_*B*_,*d*_*C*_)	Likelihood value
*n*_*xxx*_ = *n*		(0,0,0)	− *n*log(4)
*n*_*xyz*_ = *n*_*xyx*_ = *n*_*yxx*_ = 0	*n*_*xxx*_ ≤ *n*/4	(0,0,∞)	− *n*log(16)
	*n*_*xxx*_>*n*/4	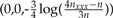	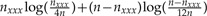
*n*_*xyz*_ = *n*_*yxx*_ = *n*_*xxy*_ = 0	*n*_*xxx*_ ≤ *n*/4	(0,∞,0)	− *n*log(16)
	*n*_*xxx*_ > *n*/4	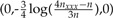	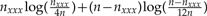
*n*_*xyz*_ = *n*_*xxy*_ = *n*_*xyx*_ = 0	*n*_*xxx*_ ≤ *n*/4	(∞,0,0)	− *n*log(16)
	*n*_*xxx*_ > *n*/4	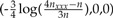	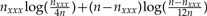

**Table 3.** Local maxima of the ML equations on the boundaries of the solution space

Conditions	Optimum	(*d*_*A*_,*d*_*B*_,*d*_*C*_)	Likelihood value
*n*_*xxx*_ + *n*_*xxy*_ > *n*/4, *n*_*xxx*_ + *n*_*xyx*_ > *n*/4	Local max	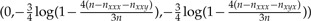	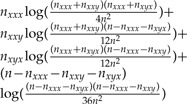
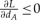			
*n*_*xxx*_ + *n*_*xxy*_>*n*/4, *n*_*xxx*_ + *n*_*yxx*_> *n*/4	Local max	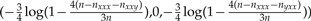	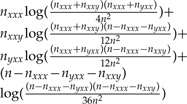
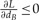			
*n*_*xxx*_ + *n*_*xyx*_>*n*/4, *n*_*xxx*_ + *n*_*yxx*_>*n*/4	Local max	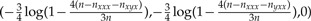	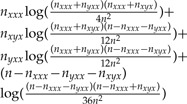
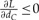			
*n*_*xxx*_ + *n*_*yxx*_> *n*/4	Local max or local min	(∞,*a*,*b*) where 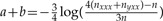	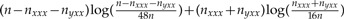
*n*_*xxx*_ + *n*_*xyx*_> *n*/4	Local max or local min	(*a*,∞,*b*) where 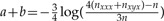	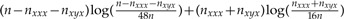
*n*_*xxx*_ + *n*_*xxy*_> *n*/4	Local max or local min	(*a*,*b*,∞) where 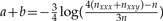	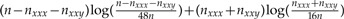
-	Local max or local min	(∞,∞,∞)	−*n*log(64)

These results correspond to the peaks at the edge of the distributions shown in Figure 3, but they do not account for the peak in the middle of the distribution, or the gap around it (clearest when *d*_*C*_ = 1.5 or 2). To explain this we need to consider the red plane, *d*_*A*_ = *d*_*B*_, in Figure 6. If we require *d*_*A*_ = *d*_*B*_, then it is possible to find an optimum which corresponds to *n*_*xyx*_ = *n*_*yxx*_. As two of the branch lengths are equal this tree is now equivalent to a three-taxon tree with a molecular clock, so the branch lengths can be derived from the solution given in Chor et al. (2006a). Examining our ML simulations shows that all of the data sets in the peak in the middle of the plots have *n*_*xyx*_ = *n*_*yxx*_, and that if *n*_*xyx*_ = *n*_*yxx*_ then the branch to C either falls exactly in the middle or on the edges of the A–B path (Supplementary Fig. 6 available on Dryad; (http://dx.doi.org/10.5061/dryad.rp7qv)). This corresponds to the optimum at *d*_*A*_ = *d*_*B*_ being either a maximum or a minimum. In comparison, if *n*_*yxx*_ and *n*_*xyx*_ differ then there are a variety of places where this branch can be placed. From this it can be deduced that the gap seen on the distribution is due to the fact that if the data are symmetric then C can either be placed in the middle or on the edge, whereas when data are not symmetric there are many more options for placement of C.

All results shown so far are for the JC model. Studies on real data generally use a more complicated model such as the general time-reversible (GTR) model (Tavaré 1986). The simulations and tree reconstructions described above have been repeated using the GTR model with realistic parameters (Murphy et al. 2001) (Supplementary Fig. 7 available on Dryad; http://dx.doi.org/10.5061/dryad.rp7qv). Again for long branch lengths many trees have zero branch lengths. However, there is no sharp peak and gap in the middle of the A–B path; we conclude that this is caused by the symmetric nature of the JC model, which is not present in the GTR model.

#### Combined ML and DM analysis

**Figure 7.**
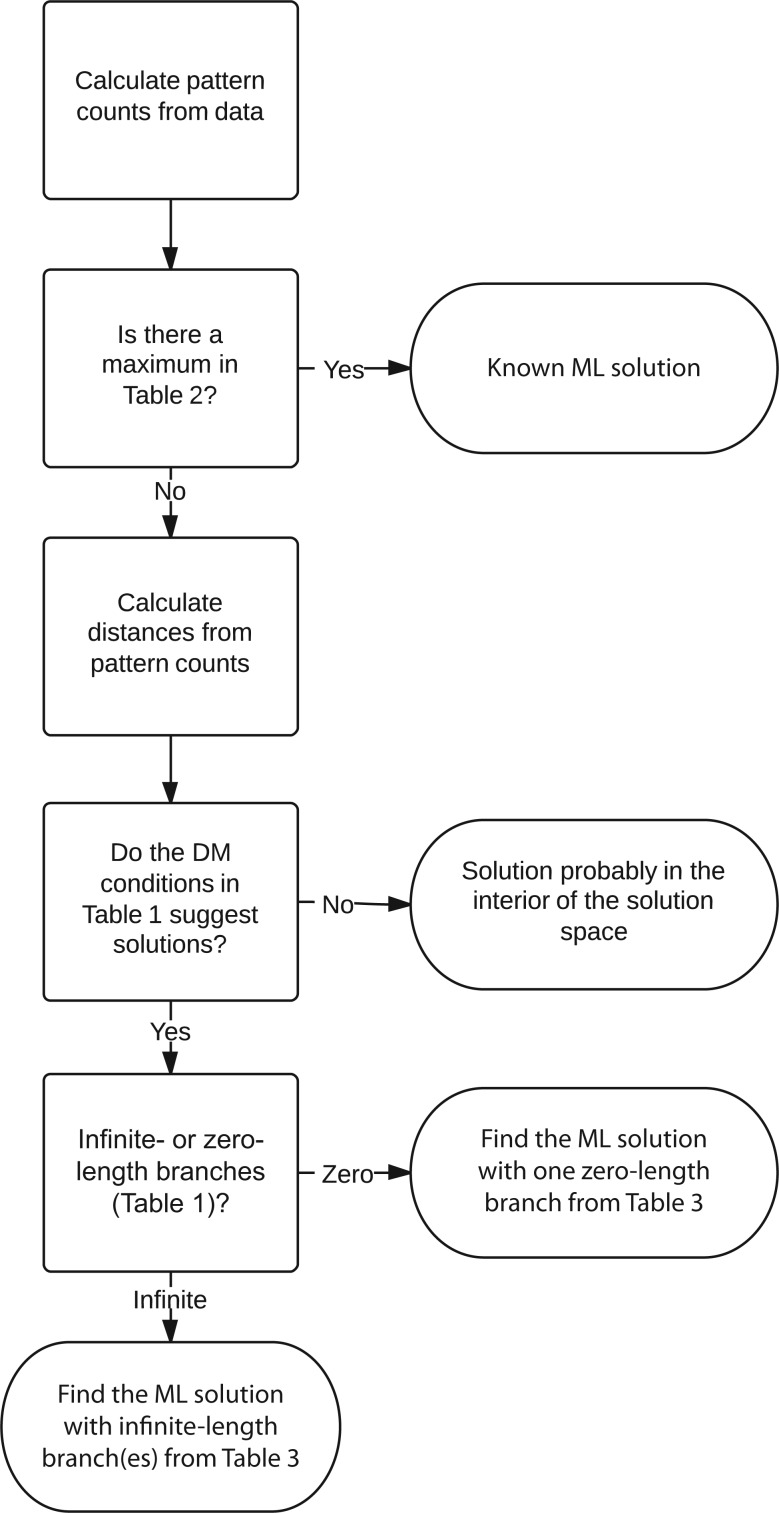
Workflow for using the ML and DM results to find the ML solution for a three-taxon tree.

Combining our ML and DM analyses allows us to gain a more complete understanding of the distributions in Figure 3. DM analysis has allowed us to predict whether the tree will have an infinite or zero branch length; in these cases, ML analysis can be used to derive the other branch lengths of the tree. Therefore, a possible workflow is as follows (Fig. 7): first, check for the known global maxima. If none of these is found, then DM analysis can be used to predict whether the tree has a zero or infinite branch length (to the described accuracy in Fig. 5). If a zero or infinite branch length is predicted, then the relevant ML solution can be used to find it. Otherwise a numerical optimization program must be used to find the global maximum.

#### Conclusions

Combining our analyses, Figure 3 can now largely be explained. This explanation can be used to split the results into separate subsets, as in Figure 4. The conditions given can be used to predict which subset a new data set will belong to (Fig. 7). An intuitive explanation can also be constructed for the trees with zero-length branches. By comparison with DM methods we can see that trees would be reconstructed with negative branch lengths. However, ML tree reconstruction does not permit negative branch lengths and hence trees are instead given zero branch lengths in these cases. These negative branch lengths are obtained because of the high variance involved in estimating long branch lengths.

We further analyzed whether the variance involved in estimating long branches could explain this phenomenon. For distance methods it is possible to estimate the variance of the estimates of *d*_*A*_, *d*_*B*_, and *d*_*C*_ as a function of the sequence length and the three branch lengths (see Supplementary Methods available on Dryad; http://dx.doi.org/10.5061/dryad.rp7qv). We are most interested in the first two of these, as these are the ones most often inferred as zero. If we assume that *d*_*A*_ is normally distributed, then it is possible to estimate the proportion of times that *d*_*A*_ is inferred to be less than or equal to zero. The same analysis can be repeated for *d*_*B*_, comparing the estimated proportions with the proportion of times that either DM or ML methods inferred that *d*_*A*_ or *d*_*B*_ was zero (Table 4). These predictions are close to the values for both DM and ML, and are slightly closer to the DM values. This is expected as they are derived from the variance of the distance estimates. The predictions tend to be slightly smaller than the proportions found in the simulations. This could be because of the approximations in the derivation of the variance (see Supplementary Methods available on Dryad; http://dx.doi.org/10.5061/dryad.rp7qv), or alternatively it could indicate that the distribution is not quite normal. This would not be surprising as, although the counts of differences between sequences may well be normally distributed, the JC distance involves a subsequent logarithmic transformation.

**Table 4.** Proportion of trees with zero branch lengths for different methods

*d*_*C*_	Predicted	Found using DM	Found using ML
0.1	0	0	0
0.5	0.0002	0	0
1	0.0224	0.0262	0.0264
1.25	0.0842	0.0998	0.1034
1.5	0.1996	0.2192	0.2202
2	0.4930	0.5064	0.5220

In summary, analysis of the variance of individual branch length estimates is able to give a good prediction of the frequency of occurrence of zero-length branches, suggesting that this could be an important explanatory factor.

### Two Long Branches

LBA is normally discussed when an (unexpected) topology with two long branches grouped together is obtained following tree reconstruction. This means LBA is generally only considered for trees with two long branches where there are multiple different possible topologies. To allow analysis of these situations, we now focus on four-taxon trees with two long branches. Two different forms of LBA have already been defined: LBC and LBJ. These will now be investigated to gain an insight into what any “attraction” might be.

#### LBC

**Figure 8.**
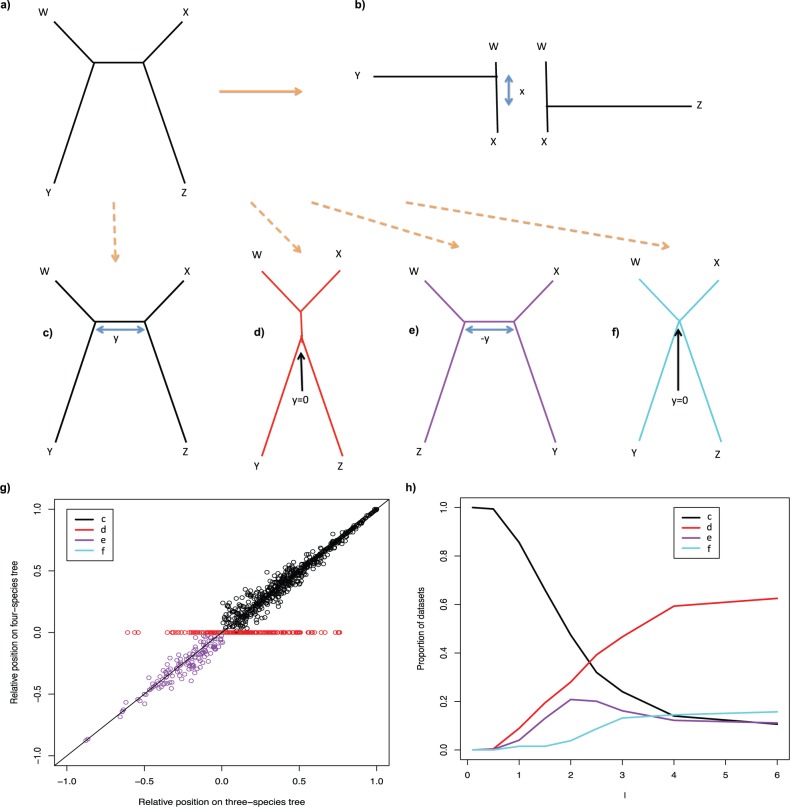
**a**) The four-taxon tree used for simulations. The path between W and X is always of length 0.1 with Y and Z evenly spaced along it. The simulated data are used to construct the ML three-taxon trees (W,X,Y) and (W,X,Z), **b**), and the ML four-taxon tree (one of **c–f**). Distances *x* and *y*, as indicated in **b–f**, measure the inferred distance between the branches to taxa Y and Z. **g**) The relative position of Y and Z on the W–X path on the three-taxon trees (*x*-axis) versus that on the optimal four-taxon tree (*y*-axis). Lengths of 1.5 are used for branches to Y and Z; equivalent results are seen for other lengths. **h**) The proportions of different topologies obtained for different lengths of Y and Z.

LBC is defined as long branches being closer together on the constructed topology than on the true topology. To investigate this we simulated four-taxon data sets from the tree in Figure 8a and applied ML to reconstruct the two three-taxon trees in Figure 8b, and the best four-taxon tree (one of Fig. 8c–f). This allows us to assess how the placement of a long branch is affected by the presence of another long branch. On the three-taxon trees only one long branch is present so no attraction could have occurred.

If there were an attraction then we would expect the long branches (Y and Z) to be closer on the four-taxon tree than on the three-taxon tree. To investigate this the relative position of Y and Z on the inferred trees has been calculated. To find the relative position on the three-taxon trees the position of the branches to Y and Z are calculated as fractions along the W–X path of their respective trees, as previously; the relative position, *x*, is then the difference between these two fractions (Fig. 8b). For each four-taxon tree the positions are again calculated for Y and Z as fractions for each topology and the relative position *y* is recorded (Fig. 8c–f). For topology 8d and 8f, *y* = 0 is recorded as the branches to Y and Z fall in the same place on the W–X path. All simulations were performed as described in “Simulations” section. The length of the W–X path is kept constant at 0.1 with Y and Z evenly spaced between W and X.

Figure 8g shows distributions of the relative position of Y and Z for the three-taxon trees (*x*-axis) against that for the four-taxon tree (*y*-axis) when the length of the branches to Y and Z is 1.5. The points are colored according to the topology of the inferred ML four-taxon tree. Also indicated is the line *x* = *y*; points on this line have the same relative position on the three- and four-taxon trees. If topology 8c, the correct topology, underwent LBC then the black points would lie below this line. Similarly, the points for topology 8e, a wrong topology with the long branches not joined to one another, would lie above this line. As can be seen these points are not distributed as would be expected for LBC; in fact there is a small asymmetry in the opposite direction to that which would be expected under LBC. This shows that the branches do not get closer together; if anything they get slightly further apart. This asymmetry becomes significant (binomial, *P* < 0.05) for topology 8c once the long branches are of length 1.5. For topology 8e this asymmetry is significant (binomial, *P* < 0.05) earlier, at a branch length of 0.75. For topologies 8c and 8e the positions of Y on the three-taxon tree are also very similar to their positions on the four-taxon tree (Supplementary Fig. 8 available on Dryad; http://dx.doi.org/10.5061/dryad.rp7qv; correlations of 0.96 and 0.97, respectively). The equivalent can be shown for the position of Z (results not shown). These results clearly show that for topologies 8c and 8e there is no attraction and no LBC occurs. This is the case for any Y and Z lengths (results not shown). We have also explored the possibility that instead of long branches becoming closer together, short branches become closer together. This can be analyzed analogously to LBC, and it can be shown that there is also no short branch closeness (results not shown).

#### LBJ

LBJ is defined as long branches being incorrectly joined to one another on a tree. To investigate this we measured the proportion of different ML topologies for different long branch lengths (Fig. 8h). For short branch lengths the results are as expected with the majority of the data sets having the correct topology. As the long branch length increases the proportion of the correct topology (8c) decreases, and the proportions of the other topologies increase, with the topology with the long branches placed together (8d) increasing in proportion more than topology 8e. For branch lengths longer than 2 topology 8d continues to increase, whereas topology 8e starts to decrease. Finally topology 8d levels off at ∼60% of the trees with all the other topologies levelling off at ∼13%. This shows that for very long branch lengths there is a strong bias toward placing the long branches together and that for infinite branch lengths instead of getting each topology chosen randomly, topology 8d would be chosen over half of the time. This shows that LBJ is occurring.

The details of these results are dependent on both sequence length and the length of the W–X path. If sequence length is increased then longer branch lengths are required to see the patterns shown here: however, with long-enough branch lengths they will still occur. However, for any length of branch to Y and Z, if sequence length is increased enough then the correct topology will be reached 100% of the time, as ML phylogenetic inference is consistent. The final proportions of the topologies are dependent on the length of the W–X path; however, the existence of the bias is not removed by changing the W–X path length.

As with the three-taxon tree problem, the simulations and tree reconstructions shown above have been repeated using the GTR model with realistic parameters (Murphy et al. 2001) (Supplementary Fig. 9 available on Dryad; http://dx.doi.org/10.5061/dryad.rp7qv). Again LBC does not occur (results not shown) but for long branch lengths LBJ does occur. However, longer branch lengths are required for LBJ to occur with GTR than with JC. This is probably because, although on average the bases are mutating at the same rates, in the GTR model some rates will be slower than average, and some faster. This means that saturation will not be reached by all sites at the same time, so at long branch lengths there will still be information about the tree in some of the sites. Connecting this with the concept of effective sequence length (Nasrallah et al. 2011), the length of an “ideal” sequence required to get the same behavior as a real sequence, indicates that effective sequence length may be model dependent. It is important to note that the comparison of GTR and JC does not tell us which model would perform better if there were any model misspecification, as would likely be the case in the majority of empirical studies.

We find the extent of the phenomenon of LBJ surprising. It is important to note that when two quantities can tend to infinity, the order in which limits to infinity are taken can be important. The extent of LBJ is affected by both the sequence length and the long branch length, and the outcome is controlled by the order in which these approach infinity. If we take *P*_*n*,*L*_(*T*′) to be the probability that ML recovers tree *T*′ (any tree, including *T*) from *n* sites generated on *T*, where *L* is the long branch length, then if we take sequence length to infinity first we obtain:

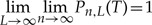

If instead we reverse the order of the limits then we obtain:

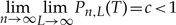

(If limits are taken simultaneously then *P*_*n*,*L*_(*T*) converges to *c* < 1 unless *n* grows exponentially faster than *L*, in which case *P*_*n*,*L*_(*T*) converges to 1 (Martyn and Steel 2012)). This convergence to a value < 1 is what we are seeing in Figure 8h, where for long branch lengths the correct tree is only obtained ∼13% of the time. To understand this phenomenon it would be useful to obtain bounds on *c*. It is possible to show that, in the limits, the probability of obtaining topology 8c and topology 8e is the same, and hence *c* ≤ 1/2 (see Supplementary Methods available on Dryad; http://dx.doi.org/10.5061/dryad.rp7qv). This is still much larger than the 13% seen in our simulation. We have not been able to obtain tighter bounds for *c*. If this were possible then it could significantly improve our understanding of LBJ.

#### Conclusions

The addition of an extra taxon to a tree increases the number of possible wrong trees which could be inferred, and stochastic error means that they will be inferred sometimes. We have shown that when long branches are not joined to one another they do not appear to attract, so there is no LBC. However, the proportion of time long branches join is dependent on branch length, and biases toward trees with long branches placed together get worse as branch lengths increase. These results show that LBJ does happen and is related to the existence of long branches, but it is caused neither by inconsistency or attraction. LBJ may be a better term than LBA.

## Conclusions

We have shown that placing one long branch is difficult for ML, even with the correct model. Counterintuitively, there is a bias toward the tips of the three-taxon tree. Application of DM and ML equations has led to insights as to why this bias exists, as well as predictions and ML solutions for trees with zero and infinite branch lengths.

LBA has been analyzed for small trees and two distinct analyzable phenomena distinguished: LBC and LBJ. LBC is defined as long branches being closer together on the constructed topology than on the true topology. LBJ is defined as long branches being incorrectly joined together on a tree. It has been shown that LBC does not exist on four-taxon trees, and that the long branches do not interact with each other when they are not placed together on a tree. However, LBJ does exist and is the same effect as found previously (Huelsenbeck and Hillis 1993). As LBC does not exist, the phrase LBA, which has come to be used for this effect, does not seem appropriate. The reason for LBJ is still an open question.

The results shown here have been obtained with long branch lengths and limited amounts of data, which raises the question of whether we are likely to see any of these effects in real data. It is difficult to make direct comparisons from the results shown here to papers citing LBA because real data will not conform to a specific evolutionary model, and is likely to be significantly more complicated than the model examined here. Additionally, empirical studies all use more than three taxa. The effects described in this article were seen for single long branches as short as 1 (expected substitution per site), well within the bounds of many existing studies. For the cases with two long branches, LBJ only becomes a real problem when the long branches are of length 2 or greater. For these lengths it would be difficult to align the sequences. However, real sequences have much more complicated evolution than that assumed here, and there is no way of dismissing LBJ as a possible problem for real data.

Previously a large number of tests for LBA have been suggested. Our results indicate that these tests may not all be appropriate. For example, one such method is based on removing one of the long branches and then repeating the reconstruction. If the long branch maintains its original position then this was taken to indicate LBA had not taken place (Pol and Siddall 2001). However, we have shown that even one long branch is not necessarily expected to be placed correctly, suggesting this test may not be enough. Another method proposes detection of LBA by comparing results using a phylogenetic inference method that suffers less from LBA (Huelsenbeck 1997), but our finding that even ML can suffer from LBA without model misspecification indicates that care should be taken to ensure methods shown to be robust to LBA are used.

Our study shows that even one long branch may be placed incorrectly and in an unexpected way by ML on problems as simple as three- or four-taxon trees with a correctly specified substitution model. Although not in itself informative about behavior on larger trees, this gives cause for concern when analyzing trees with even one very long branch, and highlights the fact that investigations involving larger trees are needed. There is still a lot that we do not understand about simple models on small trees.

## Supplementary Material

Data available from the Dryad Digital Repository: http://dx.doi.org/10.5061/dryad.rp7qv.

## Funding

The work was supported by the European Molecular Biology Laboratory (S.L.P. and N.G.). S.L.P. was also supported by the Biotechnology and Biological Sciences Research Council (BBSRC) and is a member of Sidney Sussex College, University of Cambridge.
